# Dose Dependent Effects of Fructose and Glucose on de novo Palmitate and Glycerol Synthesis in an Enterocyte Cell Model

**DOI:** 10.1002/mnfr.202100456

**Published:** 2021-12-03

**Authors:** Simon Steenson, Fariba Shojaee‐Moradie, Julie A. Lovegrove, A. Margot Umpleby, Kim G. Jackson, Barbara A. Fielding

**Affiliations:** ^1^ Department of Nutritional Sciences Faculty of Health and Medical Sciences University of Surrey Guildford GU2 7WG UK; ^2^ Hugh Sinclair Unit of Human Nutrition Department of Food & Nutritional Sciences and Institute for Cardiovascular and Metabolic Research (ICMR) University of Reading Reading RG6 6DZ UK

**Keywords:** Caco‐2, de novo lipogenesis, fructose, glucose, glycerol

## Abstract

**Scope:**

Fructose exacerbates post‐prandial hypertriacylglycerolaemia; perhaps partly due to increased enterocyte de novo lipogenesis (DNL). It is unknown whether this is concentration‐dependent or if fructose has a greater effect on lipid synthesis than glucose. Dose‐dependent effects of fructose and glucose on DNL and de novo triacylglycerol (TAG)‐glycerol synthesis are investigated in a Caco‐2 cell model.

**Methods and Results:**

Caco‐2 cells are treated for 96 h with 5, 25, or 50 mM fructose or glucose, or 12.5 mM fructose/12.5 mM glucose mix. DNL is measured following addition of [^13^C_2_]‐acetate to apical media. Separately, [^13^C_6_]‐fructose and [^13^C_6_]‐glucose are used to measure DNL and de novo TAG‐glycerol synthesis. DNL from [^13^C_2_]‐acetate is detected following all treatments, with greater amounts in intracellular than secreted (media) samples (all *p* < 0.05). DNL from [^13^C_6_]‐fructose and [^13^C_6_]‐glucose is also measurable. Intracellular synthesis is concentration‐dependent for both glucose (*p* = 0.003) and fructose (*p* = 0.034) tracers and is higher with 25 mM glucose than 25 mM fructose (*p* = 0.025). DNL from fructose and glucose is <1%, but up to 70% of de novo TAG‐glycerol is synthesized from glucose or fructose.

**Conclusion:**

Fructose is not a major source of DNL in Caco‐2 cells but contributes substantially to de novo TAG‐glycerol synthesis.

## Graphical abstract

This study treated intestinal Caco‐2 cells with three glucose and three fructose concentrations, and a glucose/fructose mixture, and traced the amount of de novo lipogenesis (DNL) and triacylglycerol (TAG)‐glycerol that was synthesised. Although glucose and fructose made a minor contribution to DNL (<1%), they provided the majority of glycerol used for TAG synthesis by Caco‐2 cells (30–70%).

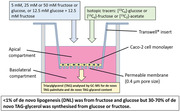

## 1 Introduction

Several studies have reported that fructose feeding in humans increases post‐prandial plasma triacylglycerol (TAG) concentrations, an independent risk factor for cardiovascular disease.^[^ ^1^, ^2^, ^3^ ^]^ Fructose may elicit this effect, in part, by increasing the synthesis and secretion of TAG from the small intestine following a high‐fat meal.^[^ ^4^ ^]^ This may be partly due to increased intestinal de novo lipogeneis (DNL). Stimulation of DNL within enterocytes in response to fructose feeding has been reported in both Syrian golden hamsters and, more recently in humans, including evidence that fructose can act as a substrate for the synthesis of new fatty acids.^[^ ^5^, ^6^ ^]^ However, when administered acutely in humans, high fructose drinks (30% of energy) did not increase intestinal DNL.^[^ ^7^ ^]^ [U^13^C]‐fructose added to a fructose test meal in humans was shown to make a major contribution to de novo TAG‐glycerol synthesis.^[^ ^8^ ^]^ This was assumed to be hepatic synthesis, but the synthesis of glycerol from fructose in the intestine has never been measured.

Caco‐2 cells are a well‐accepted and widely used in vitro model for intestinal transport studies.^[^ ^9^ ^]^ They are morphologically and physiologically similar to human enterocytes and undergo spontaneous differentiation under normal culturing conditions, forming a polarized monolayer with tight junctions between cells, as well as distinct apical and basolateral membranes. Caco‐2 cells are capable of performing the necessary apoB mRNA editing required to synthesize and secrete both apolipoprotein (apo)B100‐ and apoB48‐containing TAG‐rich lipoproteins (TRL), in response to an apical supply of free fatty acids (FFA).^[^ ^10^, ^11^, ^12^ ^]^ Furthermore, cells are known to express the sugar transporters GLUT2, GLUT5, and sodium‐glucose cotransporter 1 (SGLT1).^[^ ^13^ ^]^ We used Caco‐2 cells to determine the concentration‐dependent effects of fructose and glucose, as well as a mixture of the two sugars, on enterocyte TAG synthesis, DNL and de novo TAG‐glycerol synthesis, using stable isotope tracers.

## 2 Experimental Section

### 2.1 Cell Culture Conditions

Caco‐2 cells (passage 47) were obtained from Public Health England (European Collection of Cell Cultures [Ref: 09042001], Salisbury, UK). All materials required for cell culture experiments were obtained from Sigma‐Aldrich (UK), unless otherwise stated. Cells were maintained at 37 °C with 5% carbon dioxide (CO_2_) and 95% air with a constant 95% relative humidity in Dulbecco’s Modified Eagle’s Medium (DMEM; 25 mM glucose) supplemented with 1% (v/v) L‐glutamine, 1% non‐essential amino acids (NEAA), 1% penicillin‐streptomycin, and 20% (v/v) heat‐inactivated fetal bovine serum (FBS). Cells were passaged once they reached 80–90% confluence, as estimated using a light microscope.

Caco‐2 cells were cultured on 6‐well Transwell plates with 24 mm inserts (0.4 µm pore size, 4.67 cm^2^ area; Corning Inc., USA), on which cells grow to form a confluent and highly differentiated monolayer creating separate “apical” and “basolateral” compartments.^[^ ^14^ ^]^ Caco‐2 cells grown in this manner differentiate to form a polarized monolayer with “tight junctions,” and are capable of forming intracellular lipid droplets, as well as synthesizing and secreting both chylomicron (CM)‐ and very low‐density lipoprotein (VLDL)‐like particles.^[^ ^15^ ^]^ Cells (passage 49–55) were seeded at 5 × 10^5^ cells mL^−1^ onto the apical membrane and grown to confluence in DMEM complete medium (1.5 mL apical compartment; 2.6 mL basolateral compartment). One well on each plate was not seeded with cells and only maintained with carbohydrate (CHO)‐free medium to act as a blank reference. The integrity of the cell monolayers was assessed by transepithelial electrical resistance (TEER) measurements to provide an indication of cell differentiation and formation of “tight junctions.”^[^ ^16^ ^]^ Any wells with a TEER value of less than 300 Ω were not used. The apical and basolateral media were changed and TEER measurements were taken every 3–4 days post‐seeding. Cells reached confluence approximately 14 days after seeding. However, experiments were only initiated 21 days post‐seeding, as this extended culturing period has been shown to maximize the ability of Caco‐2 cells to synthesize and secrete TAG, with an enhanced secretion efficiency compared to 14 days post‐seeding.^[^ ^17^ ^]^ Stock solutions of glucose and fructose (250 mM) were made by dissolving each monosaccharide in CHO‐free complete DMEM (Gibco, Thermo Fisher Scientific, UK), which contained NEAA, antibiotics, L‐glutamine (all 1% v/v) and lipid depleted FBS (20% v/v), but no glucose or other source of hexose sugar. Treatment media were then prepared by diluting these stock solutions with CHO‐free complete DMEM to achieve desired molarities of 5, 25, and 50 mM.

### 2.2 Cell Viability

Before undertaking the experiments, cell viability was checked for potential cytotoxicity to ensure fructose and glucose concentrations used in experiments would not adversely affect normal Caco‐2 cell growth and metabolic activity. Cells were seeded at 5 × 10^3^ cells cm^−2^ into 12 well plates (3.9 cm^2^ well area) and grown to approximately 90% confluence for 7–8 days before treatment for a period of 96 h. A MTT (3‐(4,5‐dimethylthiazol‐2‐yl)‐2,5‐diphenyltetrazolium bromide) assay was performed to check cell viability,^[^ ^18^ ^]^ relative to 25 mM glucose, as the concentration to which cells were habituated.

### 2.3 Preparation of Fatty Acid Micelles

A fatty acid (FA) taurocholic acid (TC) micelle mixture was prepared by weighing out separate 0.269 g quantities of TC sodium hydrate (Sigma‐Aldrich, UK) into 50 mL tubes, and dissolving each in 25 mL of pre‐warmed CHO‐free complete medium containing lipid‐depleted serum (LDS) to give a concentration of 10 mmol L^−1^. Sodium palmitate and sodium stearate were each added to 1 mL of TC solution and left on a heating block for 5 min at 70 °C to dissolve, before adding to the remaining 24 mL of TC solution to give a target concentration of 5 mM. Other FAs (sodium oleate, linoleic acid sodium salt, α‐linolenic acid) were added directly into the pre‐warmed TC solutions (25 mL; 5 mmol L^−1^). All FA‐TC solutions were placed on ice and sonicated for 20–30 min (15 s pulses with a 30 s break) using a Soniprep 150 (MSE Ltd., UK) until opalescent, before shaking gently for 2 h at 37 °C to allow micelles to anneal. FA‐micelle solutions were then filter‐sterilized. To determine the final FA‐micelle concentration, each solution was diluted 1:10 with DMEM (no additions) and assayed for non‐esterified fatty acid concentration by an enzymatic method (Wako NEFA‐HR assay; Alpha Laboratories, UK) using the ILAB600 clinical chemistry analyzer. Solutions were then aliquoted and stored at −20 °C until needed. The different FA‐TC solutions were added to the treatment media to yield a final FA concentration of 0.5 mM (0.138 mM sodium palmitate (16:0), 0.065 mM sodium stearate (18:0), 0.198 mM sodium oleate (18:1 n‐9) and sodium salts of 0.083 mM linoleic acid (18:3 n‐3) and 0.017 mM alpha‐linolenic acid (18:3 n‐3)), as described previously, as a means to provide FA in a form similar to that of post‐digestion duodenal micelles in vivo.^[^ ^17^ ^]^ The final FA composition in the treatment media was designed to reflect the typical UK dietary fat intake.^[^ ^19^, ^20^ ^]^

### 2.4 Cell Treatments

Cell monolayers were washed with 1 mL of CHO‐free DMEM (with no additions) prior to treatment for 96 h. Cells were treated for 96 h with media containing the following hexose concentrations: 5 mM glucose (5G), 25 mM glucose (25G), 50 mM glucose (50G), 5 mM fructose (5F), 25 mM fructose (25F), 50 mM fructose (50F), or 12.5 mM glucose and 12.5 mM fructose (G/F Mix) (**Table** **1**). Three independent replicates were performed for each experiment. In order to measure the total amount of de novo palmitate synthesized in response to each treatment, [^13^C_2_]‐acetate (100% enrichment) was added to the treatment media to achieve a final concentration of 5 mM. In a separate set of experiments, [^13^C_2_]‐acetate was replaced by [^13^C_6_]‐glucose or [^13^C_6_]‐fructose (20% tracer, 80% unlabeled hexose; e.g., 1 mM [^13^C_6_]‐glucose for the 5 mM glucose treatment), to trace the synthesis of each sugar into de novo palmitate in TAG, as well as incorporation into the glycerol moiety of TAG. Two experiments with the 12.5 mM glucose/12.5 mM fructose mix (G/F Mix) were performed, one with [^13^C_6_]‐fructose tracer (Mix F) and one with [^13^C_6_]‐glucose tracer (Mix G).

**Table 1:** Composition of fructose and glucose treatment media used for Caco‐2 experiments

No.	Sugar type and concentration	LDS (% v/v)	FA‐TC micelle mixture [mmol L^−1^]	Stable isotope tracer
1	Glucose 5 mM	20	0.5	[^13^C_6_]‐glucose or [^13^C_2_]‐acetate
2	Glucose 25 mM	20	0.5	[^13^C_6_]‐glucose or [^13^C_2_]‐acetate
3	Glucose 50 mM	20	0.5	[^13^C_6_]‐glucose or [^13^C_2_]‐acetate
4	Fructose 5 mM	20	0.5	[^13^C_6_]‐fructose or [^13^C_2_]‐acetate
5	Fructose 25 mM	20	0.5	[^13^C_6_]‐fructose or [^13^C_2_]‐acetate
6	Fructose 50 mM	20	0.5	[^13^C_6_]‐fructose or [^13^C_2_]‐acetate
7	Glucose 12.5 mM + Fructose 12.5 mM	20	0.5	[^13^C_6_]‐fructose or [^13^C_6_]‐glucose or [^13^C_2_]‐acetate

LDS, lipid depleted fetal bovine serum.

Both intracellular and secreted TAG were collected during the treatment period and analyzed by gas chromatography‐mass spectrometry (GC‐MS) to determine the [^13^C]‐palmitate and [^13^C_3_]‐glycerol content. During the 96 h treatment period, TEER measurements were taken every 24 h to ensure monolayer integrity was maintained (≥300 Ω), prior to collecting basolateral media from each well at 24, 48, 72, and 96 h (total volume for all timepoints = 10.8 mL) and replaced with CHO‐free delipidated serum (DLS) medium. Basolateral media from each treatment were pooled (2.6 mL per well 24 h^−1^; total volume 10.4 mL) and frozen at −20 °C, before being lyophilized to dryness using a freeze drier (ModulyoD, Thermo Fisher Scientific, UK). Samples were then re‐constituted in 0.5 mL of di H_2_O prior to lipid extraction (see next section). Treatment media in the apical compartment was also changed every 24 h. Cell monolayers were harvested from each insert at the end of the treatment period. Monolayers were washed twice with ice‐cold phosphate‐buffered saline (PBS) and Transwell plates placed on ice, after which 200 µL of ice‐cold lysis buffer (Cell Signaling Technology Inc., USA) was added directly to the monolayers, before scraping with a cell scraper and transferring to an Eppendorf tube. Samples were frozen (−20 °C) until analyzed.

### 2.5 Analytical Methods

Total cell protein concentration (mg mL^−1^ cell lysate) was determined by the Bicinchoninic acid assay (Pierce BCA protein assay kit, Thermo Fisher Scientific, UK). The TAG content and isotopic enrichment of both intracellular (cell lysate; volume made up to 0.5 mL) and basolateral (cell media) samples were analyzed by extracting the total lipid content (0.5 mL sample volume) with chloroform‐methanol solution (2:1 ratio, v:v). TAG was separated from other lipid classes in the samples by thin‐layer chromataography (TLC) and hydrolyzed in the presence of 3% hydrochloric acid:methanol to yield separate layers containing glycerol from TAG (TAG‐glycerol) and fatty acid methyl esters (FAME), as previously described.^[^ ^21^ ^]^ The palmitic acid methyl ester (PAME) was analyzed via GC‐MS in order to determine the [^13^C]‐enrichment, which was used to calculate the amount of de novo TAG‐palmitate formed in response to each treatment. The isotopic enrichment of PAME was measured by selective monitoring of 9 ions: *m/z* = 270 (unlabeled palmitate; M+0), *m/z* = 272 (M+2), *m/z* = 274 (M+4), *m/z* = 276 (M+6), *m/z* = 278 (M+8), *m/z* = 280 (M+10), *m/z* = 282 (M+12), *m/z* = 284 (M+14), and *m/z* = 286 (M+16). TAG‐palmitate concentration was measured by GC‐MS by reference to an internal standard, heptadecanoate (50 µg per sample), added to samples prior to derivatization. TAG concentration was calculated from the total PAMEs divided by a factor of three.

The aqueous layer from the TAG hydrolysis step above was purified by ion exchange chromatography and derivatized to yield the tert.‐butyldimethylsilyl derivative of TAG‐glycerol and enrichment was measured by GC‐MS (Agilent 5975) in electron ionization mode. Ions monitored were *m/z* 377 and *m/z* 380 (m+3).^[^ ^22^ ^]^ The [^13^C_3_]‐glycerol enrichment of TAG‐glycerol extracted from intracellular and basolateral samples was measured to determine the utilization of fructose and glucose as substrates for TAG‐glycerol synthesis.

### 2.6 Calculations

The TTR of all enriched palmitate isotopomers relative to the abundance of unenriched palmitate (M+0) was calculated (M+2/M+0, M+4/M+0…M+16/M+0). These TTR values represented the relative [^13^C]‐enrichment in palmitate molecules that had incorporated a minimum of one (M+2/M+0) to a maximum of eight (M+16/M+0) [^13^C_2_]‐acetate subunits during palmitate synthesis. Natural “background” enrichment, determined from experiments conducted without any isotopic tracer added to the media, was subtracted from these TTR values. The number of labeled carbons in each isotopomer were summed and divided by the total number of palmitate carbons, as described previously,^[^ ^23^ ^]^ to give the total enrichment. This was multiplied by the TAG‐palmitate concentration of each sample, corrected for cell protein, to obtain the amount of de novo palmitate (pmol mg^−1^ cell protein) derived from each tracer ([^13^C_2_]‐acetate, [^13^C_6_]‐fructose, or [^13^C_6_]‐glucose). Percent de novo TAG‐glycerol was determined as:

(1) $$\mathrm{De}\;\mathrm{novo}\;\mathrm{TAG}-\mathrm{glycerol}\;\left(\%\right)=\frac{\left(\mathrm{TTR}\;\mathrm{of}\;\mathrm{TAG}-\mathrm{glycerol}\right)}{\left(\mathrm{TTR}\;\mathrm{treatment}\;\mathrm{media}\right)}\times 100$$

where the TTR of TAG‐glycerol in a sample represents the ratio of *m/z* 380/377 (corrected for natural abundance) and the TTR of [^13^C_6_]‐glucose or [^13^C_6_]‐fructose in treatment media was 0.2 (20 %). Percent de novo TAG‐palmitate from the glucose and fructose tracers was calculated with the substitution of TTR of TAG‐palmitate in the above equation.

### 2.7 Statistical Analysis

All statistical analyses were performed using SPSS version 24 (IBM Corp., USA). Data for pretreatment TEER values (days 4–21), TAG concentration, de novo palmitate and de novo TAG‐glycerol, were all analyzed via one‐way ANOVA. Separate tests were conducted to compare differences between the three glucose and the three fructose conditions (5, 25, or 50 mM), both for intracellular (cell lysate) and secreted (cell media) samples (four tests). Where variances between groups were homogenous (Levene's test *p* > 0.05), the ANOVA result was interpreted and Tukey's HSD post hoc tests used to determine significant pairwise comparisons. If variances were heterogeneous (Levene's test *p* < 0.05), a modified Welch ANOVA was conducted and followed with Games‐Howell post hoc tests.

Independent‐samples *t*‐tests were run to compare secreted and intracellular data for each treatment condition (e.g., 5 mM glucose secreted vs intracellular conditions), as well as for comparison of equivalent glucose and fructose concentrations (e.g., 5 mM glucose vs 5 mM fructose). All data are presented as mean ± SD. For all statistical tests, *p* < 0.05 was considered statistically significant.

## 3 Results

### 3.1 Cell Viability

Cell viability was shown to be greater than 85% for all glucose (5 and 50 mM) and fructose concentrations (5, 25, 50 mM), relative to the 25 mM glucose as the control condition, indicating a lack of cytotoxicity for the treatment concentrations used in experiments (data not shown).

#### 3.1.1 Transepithelial Electrical Resistance (TEER) Measurements to Assess Caco‐2 Cell Monolayer Integrity

TEER measurements consistently increased between day 4 and day 21 post‐seeding, indicating continuing differentiation and maturation of cell monolayers (data not shown). Prior to commencement of the 96 h treatment period (day 21), there were no significant differences in TEER measurements between monolayers (*p* = 0.887). TEER values for all monolayers remained above the minimum acceptable value of 300 Ω throughout the treatment period, indicating monolayer tight junction integrity was maintained.^[^ ^24^ ^]^

#### 3.1.2 Intracellular and Secreted TAG Concentration

**Figure 1:**
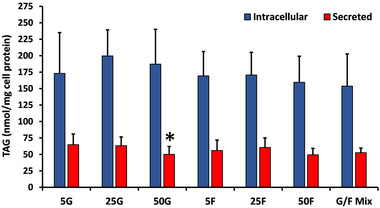
Effect of glucose (G) and fructose (F) concentration (mM) on the TAG content of cells (intracellular) and basolateral media (secreted). Data are mean ± SD for *n* = 9–18 independent experiments. For all fructose and glucose concentrations, intracellular TAG content was significantly higher than the secreted TAG (all *p* < 0.0005; independent samples *t* tests). The effect of the different glucose and fructose conditions on secreted and intracellular TAG were analyzed separately by one‐way ANOVA (Tukey's *post hoc*); *p = 0.035 for secreted TAG versus 5G. Abbreviations: G/F Mix, 12.5mM G + 12.5 mM F.

For all fructose and glucose concentrations, the intracellular TAG content was significantly higher than the secreted TAG (all *p* < 0.0005) (**Figure** **1**). There were no significant differences in TAG concentration between the three glucose or fructose concentrations, except for secreted TAG following 50 mM glucose (50.1 ± 11.9 nmol mg^−1^ cell protein; mean ± SD), which was lower than 5 mM glucose (64.8 ± 16.1 nmol mg^−1^ cell protein) (*p* = 0.035). There were no significant differences between equivalent glucose and fructose concentrations for either intracellular or secreted TAG.

#### 3.1.3 De novo TAG‐palmitate Synthesized from [^13^C_2_]‐acetate

**Figure 2:**
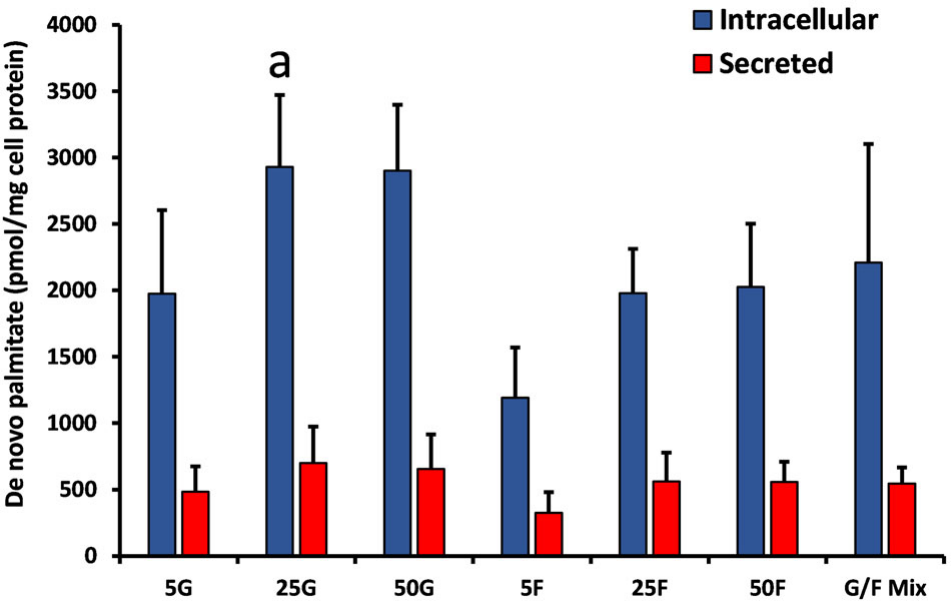
Effect of glucose (G) and fructose (F) concentration on de novo TAG‐palmitate synthesis from [^13^C_2_]‐acetate. Data are mean ± SD for *n* = 4–5 independent experiments. The effect of concentration on intracellular and secreted de novo palmitate was analyzed separately for glucose and fructose treatments by one‐way ANOVA (NS). For all fructose and glucose concentrations, de novo TAG‐palmitate was significantly higher in intracellular than secreted samples (all *p* < 0.05; independent samples *t*‐tests). Differences between glucose and fructose for each concentration were analyzed by separate independent‐samples *t*‐tests. **a** *P* < 0.05 versus intracellular 25F. Abbreviations: G/F Mix, 12.5mM G + 12.5 mM F.

De novo palmitate synthesis from [^13^C_2_]‐acetate was between 3.5 and 4.4‐fold higher in intracellular than secreted (media) samples for all treatments (all *p* < 0.05) (**Figure** **2**). There were no significant effects of dose within the glucose or fructose conditions, either for secreted or intracellular de novo TAG‐palmitate. When comparing the sugars, intracellular de novo TAG‐palmitate with 25 mM glucose was higher than with 25 mM fructose (*p* < 0.05).

#### 3.1.4 De novo TAG‐palmitate Synthesized from [^13^C_6_]‐fructose or [^13^C_6_]‐glucose

**Figure 3:**
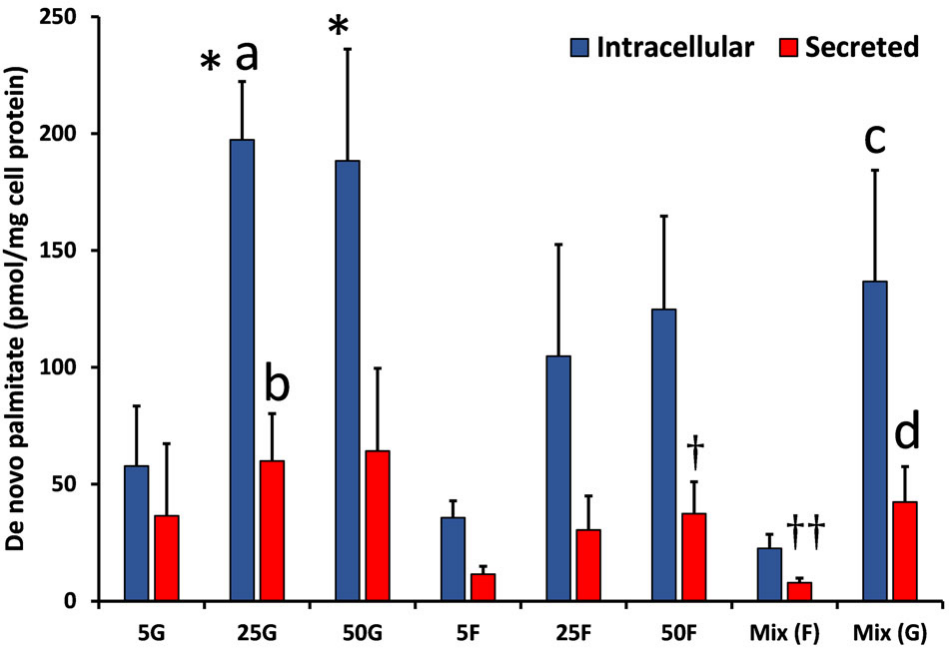
Effect of glucose (G) and fructose (F) concentration (mM) on de novo TAG‐palmitate synthesis from [^13^C_6_]‐fructose or [^13^C_6_]‐glucose. Data are mean ± SD for *n* = 4–5 independent experiments. The effect of concentration on intracellular and secreted de novo TAG‐palmitate was analyzed separately for glucose and fructose treatments by one‐way ANOVA (Tukey's *post hoc*). For intracellular glucose groups; * *p* < 0.01 versus 5G. For secreted fructose groups; **†** *p* < 0.05 versus 5F, **††** *p* < 0.01 versus 25F and 50F. Differences between equivalent glucose and fructose concentrations were analyzed by independent‐samples *t* test; **a** *p* < 0.05 versus intracellular 25F, **b** *p* < 0.05 versus secreted 25F. **c** *p* < 0.01 versus intracellular Mix (F); **d** *p* = 0.01 versus secreted Mix (F). Abbreviations: Mix (F), 12.5G + 12.5F ([^13^C_6_]‐fructose tracer); Mix (G), 12.5G + 12.5F ([^13^C_6_]‐glucose tracer).

De novo TAG‐palmitate synthesis from [^13^C_6_]‐fructose or [^13^C_6_]‐glucose was measurable for all treatment conditions (**Figure** **3**) and was greater for intracellular than for secreted samples (all *p* < 0.05; with the exception of 5 mM glucose, *p* = 0.57). Intracellular synthesis was dose‐dependent for both glucose and fructose tracers (ANOVA: *p* = 0.003, *p* = 0.034, respectively), and was almost 2‐fold higher with 25 mM glucose (197 pmol mg^−1^ cell protein) than 25 mM fructose (105 pmol mg^−1^ cell protein; *p* < 0.03). Secreted synthesis was dose dependent for fructose treatments (ANOVA, *p* = 0.002) but not glucose treatments, and was higher for 25 mM glucose than 25 mM fructose (*p* < 0.05). Percentage de novo TAG‐palmitate synthesis from fructose and glucose was <1% (data not shown).

Comparison of the Mix (F) and Mix (G) conditions revealed a greater amount of de novo TAG‐palmitate synthesized from [^13^C_6_]‐glucose than from [^13^C_6_]‐fructose in both the intracellular (*p* < 0.01) and secreted samples (*p* = 0.01).

#### 3.1.5 Percent de novo TAG‐glycerol Synthesized from [^13^C_6_]‐fructose or [^13^C_6_]‐glucose

**Figure 4:**
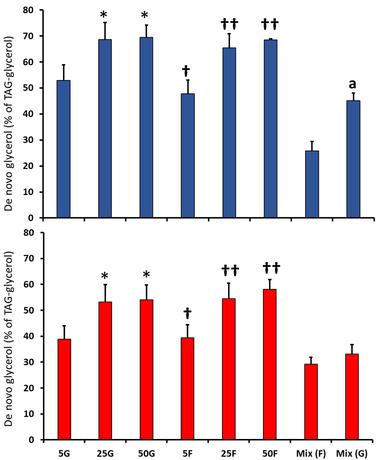
Effect of glucose (G) and fructose (F) concentration (mM) on the percentage of intracellular (upper panel) and secreted (lower panel) TAG‐glycerol synthesized from [^13^C_6_]‐fructose or [^13^C_6_]‐glucose. Abbreviations: Mix (F), 12.5G + 12.5F ([^13^C_6_]‐fructose tracer); Mix (G), 12.5G + 12.5F ([^13^C_6_]‐glucose tracer). The effect of concentration was analyzed separately for glucose and fructose treatments by one‐way ANOVA (Tukey's *post hoc*). Differences between equivalent glucose and fructose concentrations were analyzed by independent‐samples *t* test. Intracellular treatments: For glucose groups; * *p* < 0.05 versus 5G and *p* < 0.0005 versus Mix (G). For fructose groups; **†** *p* < 0.0005 versus Mix (F), **††** *p* < 0.005 versus 5F and *p* < 0.0005 versus Mix (F); a *p* < 0.0005 versus Mix (F). Secreted treatments: for glucose groups; *p < 0.01 versus 5G and *p* < 0.0005 versus Mix (G). For fructose groups; **†** *p* < 0.05 versus Mix (F), **††** *p* < 0.0005 versus 5F and *p < *0.0005 versus Mix (F).

Between 30% and 70% of de novo TAG‐glycerol was synthesized from glucose or fructose, with no difference in percent contribution from the two hexoses (**Figure** **4**). Both intracellular and secreted synthesis was dose‐dependent for glucose (ANOVA: *p* < 0.001; *p* < 0.001 respectively) and fructose (ANOVA: *p* < 0.001; *p* < 0.0001 respectively).

In a fructose/glucose mixture, glucose was the preferred substrate for intracellular de novo TAG‐glycerol (*p* < 0.0005). This difference was not found for secreted de novo TAG‐glycerol.

## 4 Discussion

To our knowledge, this is the first study to measure the contribution of fructose and glucose to DNL and de novo‐glycerol synthesis in Caco‐2 cells. We showed that Caco‐2 cells synthesized TAG‐palmitate de novo directly from acetate and also from glucose and fructose, an indirect pathway via acetate. The synthesis of de novo TAG‐palmitate from glucose tended to be higher than from fructose. Glucose and fructose made only a minor contribution to de novo TAG‐palmitate synthesis but made a substantial dose‐dependent contribution to the synthesis of de novo TAG‐glycerol, ranging from 30% to 70%. Percent de novo TAG‐glycerol was not different when the cells were incubated with equimolar glucose or fructose alone. However, glucose was the preferred substrate for intracellular de novo TAG‐glycerol in an equimolar mixture of the two sugars.

All treatments used in the current study resulted in approximately three‐ to four‐fold higher intracellular than secreted TAG content. This finding is consistent with values reported in Caco‐2 cells in other studies,^[^ ^15^, ^25^ ^]^ and this likely reflects a relatively limited capacity for Caco‐2 cells to synthesize and secrete TRL, compared to human enterocytes in vivo. It is also possible that treatment for 96 h may have resulted in feedback inhibition of lipid secretion by secreted chylomicron‐like particles on the basolateral side, although samples were collected at 24 h intervals during the treatment period, and so this would have reduced the chance of this occurring. Surprisingly, a significantly higher secretion of TAG was observed in the current study in response to 5 mM glucose than 50 mM glucose. This greater secretion of TAG in response to a lower apical glucose concentration has been reported previously by Pauquai et al.,^[^ ^26^ ^]^ in the only other published study to date investigating the influence of an altered carbohydrate concentration on Caco‐2 TAG metabolism. Lou^[^ ^27^ ^]^ cultured Caco‐2 cells under conditions of either high (25 mM) or low (5 mM) glucose, in both the apical and basolateral compartments, and found no significant differences in the intracellular or secreted TAG concentration. This finding was irrespective of whether the media was supplemented with 0.5 mM oleic acid or not, and supports the results of the current study. However, in the latter study, the addition of oleic acid to the media was associated with a 2.2‐ to 3.0‐fold increase in cellular and basolateral TAG for both high and low glucose conditions. These data, taken together with those of Jackson et al.^[^ ^25^ ^]^ and Bateman et al.,^[^ ^15^ ^]^ suggest the presence and type of fatty acid in the cell medium is a more important determinant of TAG synthesis and secretion by Caco‐2 cells than the glucose or fructose concentration.

We were unable to measure the acetate precursor pool used for DNL synthesis, and thus were unable to determine the precursor pool enrichment, and so chose to express de novo palmitate synthesis from the [^13^C_2_]‐acetate tracer as an absolute amount in terms of pmol, rather than as % of total DNL. It has previously been shown that labelled acetate is substantially diluted intracellularly, including by acetate produced from pyruvate (the end product of glycolysis).^[^ ^28^ ^]^ For the glucose and fructose tracer we assumed minimal intracellular dilution and showed % de novo palmitate synthesis from these tracers was <1%. This suggests there must be a high contribution to DNL from other sources, as suggested in a review of human metabolic tracer studies which estimated that less than 1% of dietary fructose was directed toward DNL.^[^ ^29^ ^]^ We recently compared the effect of high fructose drinks (30% of energy) with low fructose drinks (<0.2% energy), administered acutely, on intestinal DNL in humans.^[^ ^7^ ^]^ This study provided the first measurement of the rate of intestinal DNL in humans. Although the high fructose intake increased plasma TAG, intestinal DNL was not affected. The small increase in % DNL with fructose in our Caco‐2 cell model supports our in vivo finding.

The minor increase in DNL contrasts with the substantial contribution of glucose and fructose to de novo glycerol synthesis. When glucose was the tracer, % de novo glycerol was not dissimilar to that shown by Collins et al.^[^ ^30^ ^]^ in differentiating human adipocytes, where glucose was shown to provide 72% of the carbon of TAG‐glycerol. The enzymes for glycerol‐3‐phosphate synthesis from glucose and fructose are abundantly expressed in enterocytes.^[^ ^31^ ^,32]^ Caco‐2 cells may differ from human enterocytes in their requirement for glycerol for TAG synthesis. Due to a low MGAT activity, Caco‐2 cells primarily synthesize TAG through the glycerol 3‐phosphate acyltransferase (GPAT) pathway.^[^ ^33^ ^]^ This contrasts with enterocytes in vivo. The main pathway of TAG synthesis in the intestine is considered to be via monoacylglycerol acyltransferase (MGAT).^[^ ^34^ ^]^ Non‐esterified fatty acids and monoacylglycerol are taken up from mixed micelles into enterocytes and re‐esterified to diacylglycerol (DAG) by the action of MGATs and subsequently to TAG by DGATs. However, a recent study in mice has shown that deletion of MGAT does not affect lipid absorption,^[^ ^35^ ^]^ whereas deletion of GPAT3 results in abnormalities in dietary lipid absorption, demonstrating that this pathway may be of more importance in vivo than previously recognized.^[^ ^36^ ^]^ GPAT3 is abundantly expressed in the apical surface of enterocytes with regional localization in the jejunum; the primary site of lipid absorption.

Other sources of carbon for glycerol synthesis include amino acids, in particular glutamine (a component of the treatment media), an important energy source in enterocytes.^[^ ^37^ ^]^ Phosphoenolpyruvate carboxykinase (a key gluconeogenic enzyme) is expressed in the enterocytes of the small intestine, and deletion of this gene in mice decreases intestinal TAG secretion and reduces the catabolism of glutamine, suggesting the gluconeogenic pathway is an important source of glycerol 3‐phosphate,^[^ ^38^ ^]^ and provides further evidence that the GPAT pathway is of major importance in TAG synthesis.

In vivo, the intestine has been shown to take up large quantities of glucose from the circulation,^[^ ^39^ ^]^ some of which could be used for the synthesis of TAG‐glycerol. Glycerol can also be taken up by the intestine from the circulation and has been recovered in chylomicron TAG following i.v. administration of D_5_‐glycerol.^[^ ^40^ ^]^

While it is known that intestinal cells utilize glucose for TAG‐glycerol synthesis, our study clearly demonstrates the in vitro capacity of Caco‐2 cells to also metabolize fructose. Interestingly, a recent study in mice has shown the intestine helps to shield the liver from fructose.^[^ ^41^ ^]^ The enzyme ketohexokinase (KHK), which is substantially expressed in the small intestine, converts fructose to fructose‐1‐phosphate, a rate limiting step in fructose metabolism, and drives fructose conversion to other metabolites, including glucose and lactate. Deletion of the gene for this enzyme in the intestine only resulted in increased fructose‐induced lipogenesis in the liver.

A limitation of our study is that we did not assess whether the concentrations of fructose and glucose used had any effect on the osmolarity of the treatment media. More generally, while a widely utilized cell line, there are several differences between differentiated Caco‐2 cells and mature human enterocytes and we caution against directly extrapolating our findings to an in vivo context.

Further research is needed to determine how much glucose and fructose is released into the basolateral media. This could provide further useful additional information regarding the utilization of hexose sugars by Caco‐2 cells. Measurement of gene expression for glucose and/or fructose transporters (e.g., GLUT5), fructolytic (e.g., KHK), glycolytic, or lipogenic enzymes (e.g., fatty acid synthase), or expression of apo‐B48 in response to the treatments, could also give valuable mechanistic insights into the effects of different fructose and glucose concentrations on Caco‐2 TAG metabolism.

In conclusion, this study provides evidence that Caco‐2 cells are capable of utilizing both fructose and glucose as metabolic substrates for de novo synthesis of fatty acids and TAG‐glycerol, although neither fructose nor glucose was a major source of DNL in our enterocyte model. This suggests that increased DNL from fructose is unlikely to be a contributory factor for increased postprandial hypertriglyceridemia following fructose feeding. However, we found that both fructose and glucose make a major contribution to de novo TAG‐glycerol synthesis.

## Conflict of Interest

The authors declare no conflict of interest.

## Author Contributions

K.G.J. and B.A.F. are senior authors. M.U., B.F., J.A.L., and K.G.J. designed the experiments. S.S. performed all the experiments under the supervision of K.G.J., B.F., M.U., and F.S.M. S.S. analyzed and interpreted the data under the supervision of M.U. and B.F. M.U. and S.S. drafted the manuscript. All authors contributed to the final manuscript and approved its final version.

## Data Availability

The data that support the findings of this study are available from the corresponding author upon reasonable request.
